# Heavily treatment-experienced people living with HIV in the OPERA® cohort: population characteristics and clinical outcomes

**DOI:** 10.1186/s12879-023-08038-w

**Published:** 2023-02-13

**Authors:** Ricky K. Hsu, Jennifer S. Fusco, Cassidy E. Henegar, Vani Vannappagari, Andrew Clark, Laurence Brunet, Philip C. Lackey, Gerald Pierone, Gregory P. Fusco

**Affiliations:** 1 NYU Langone Health Center, New York, NY USA; 2 AIDS Healthcare Foundation, New York, NY USA; 3 Epividian, Inc., Raleigh, NC USA; 4 ViiV Healthcare, Durham, NC USA; 5 ViiV Healthcare, Brentford, Middlesex, UK; 6 Wake Forest School of Medicine, Winston-Salem, NC USA; 7 Whole Family Health Center, Vero Beach, FL USA; 8 Epividian, Inc., 150 Fayetteville Street, Suite 2300, Raleigh, NC 27601 USA

**Keywords:** HIV, ART, HTE, Prevalence, Characteristics, Outcomes

## Abstract

**Background:**

Multi-class resistance, intolerance, and drug–drug interactions can result in unique antiretroviral (ART) combinations for heavily treatment-experienced (HTE) people living with HIV (PLWH). We aimed to compare clinical outcomes between HTE and non-HTE PLWH.

**Methods:**

Eligible ART-experienced PLWH in care in the OPERA® Cohort were identified in a cross-sectional manner on December 31, 2016 and observed from the date of initiation of the ART regimen taken on December 31, 2016 until loss to follow up, death, study end (December 31, 2018), or becoming HTE (non-HTE group only). In the absence of resistance data, HTE was defined based on the ART regimens used (i.e., exposed to ≥ 3 core agent classes or regimen suggestive of HTE). Time to virologic undetectability, failure, and immunologic preservation were assessed using Kaplan–Meier methods; cumulative probabilities were compared between the two groups. Regimen changes, incident morbidities, and death were described.

**Results:**

A total of 24,183 PLWH (2277 HTE PLWH, 21,906 non-HTE) were followed for a median of 28 months (IQR 21, 38). Viremic HTE PLWH (viral load [VL] ≥ 50 copies/mL) were less likely to achieve undetectability (VL < 50 copies/mL; 24-month cumulative probability: 80% [95% Confidence Interval 77–82]) than their non-HTE counterparts (85% [84–86]). No difference was observed in the probability of maintaining VLs < 200 copies/mL over the first 48 months after achieving suppression (< 50 copies/mL). HTE PLWH were less likely than non-HTE PLWH to maintain CD4 cell counts ≥ 200 cells/µL (24-month cumulative probability: 95% HTE [91–93]; 97% non-HTE [97–97]), and more likely to change regimens (45% HTE; 41% non-HTE). Incident non-AIDS defining event (ADE) morbidities were common in both populations, though more likely among HTE PLWH (45%) than non-HTE PLWH (35%). Incident ADE morbidities and deaths were uncommon among HTE (ADEs 5%; deaths 2%) and non-HTE (ADEs 2%; deaths 1%) PLWH.

**Conclusions:**

HTE PLWH were at greater risk of unfavorable treatment outcomes than non-HTE PLWH, suggesting additional therapeutic options are needed for this vulnerable population.

**Supplementary Information:**

The online version contains supplementary material available at 10.1186/s12879-023-08038-w.

## Background

The development of antiretroviral therapy (ART) has prolonged life expectancy for people living with HIV (PLWH) [1]. However, long-term exposure to ART can eventually lead to fewer therapeutic options in multiple classes of ART [2, 3], due to multiple factors including poor tolerability, drug toxicity, avoidance of drug–drug interactions, and viral resistance [3–6]. As a result, a subset of PLWH require highly tailored ART regimens with less common combinations of antiretrovirals (ARV); these individuals are often referred to as heavily treatment experienced (HTE) PLWH [7].

HTE prevalence varies depending on the setting and the definition used. In the United States (US), HTE prevalence has been estimated to range from 2 to 14% in 2016–2017 [8, 9]. In Europe, HTE prevalence increased from 6% in 2010 to 9% in 2016; important geographic variation has also been observed, from 1% in Eastern Europe to 16% in Western/Central Europe [10]. The prognosis for HTE PLWH appears to be less favorable compared to less experienced PLWH. Increased risks of death and AIDS defining events (ADE) have been reported among PLWH with multidrug resistance [7, 11, 12]. Moreover, each additional ART regimen failed in the past has been associated with a higher rate of viral rebound (i.e., two consecutive viral loads (VL) > 400 copies/mL or one VL > 400 copies/mL followed by initiation of at least 2 new ARVs) [13].

Complex ART regimens, which are characteristic of treatments for HTE PLWH, may lack sufficient efficacy and have adverse safety and tolerability profiles, making disease and toxicity management even more difficult [14–16]. Though the development of new ARVs has increased the number of treatment options available for HTE PLWH, there are still individuals who cannot achieve complete virologic suppression and remain at risk for disease progression [16]. Given the scarcity of studies reporting on treatment outcomes in this population, the objective of this study was to describe the clinical outcomes and treatment management of HTE PLWH, including a depiction of the potential level of virologic control, immunologic response, and expected regimen durability in this late-stage setting.

## Methods

### Study population and design

The Observational Pharmacoepidemiology Research and Analysis (OPERA®) cohort is a clinical cohort utilizing electronic health record (EHR) data in the US and Puerto Rico. All data reflect routine medical care, with visits and testing scheduled at the discretion of the treating providers. Information captured in the EHR system at each site is retrieved, cleaned, aggregated, and anonymized to maintain patient confidentiality. OPERA® complies with all Health Insurance Portability and Accountability Act (HIPAA) and Health Information Technology for Economic and Clinical Health Act (HITECH) requirements; security and privacy rules to implement technical safeguards for the protection of electronic healthcare information and disclosure of data breaches in the US. OPERA® has received annual institutional review board (IRB) approval by Advarra IRB, including a waiver of informed consent and authorization for use of protected health information.

**Fig. 1:**
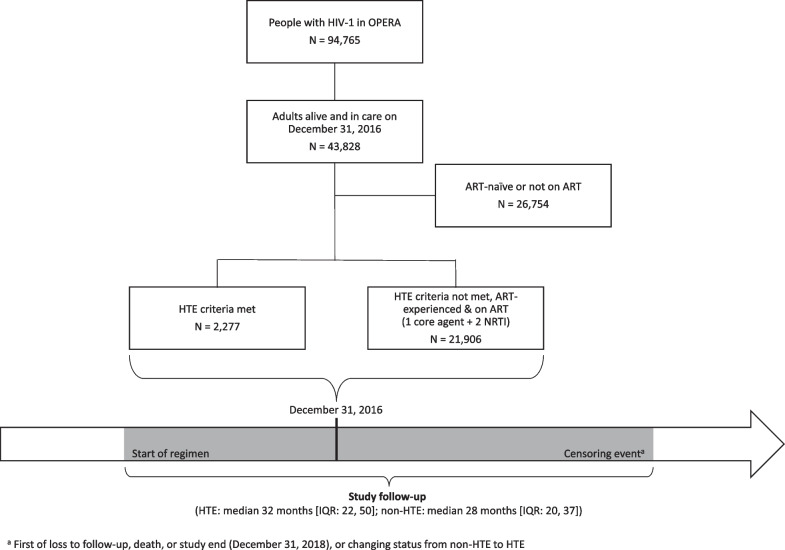
Inclusion into the study population and study timeline

The study population (Fig. 1) was identified in a cross-sectional manner and consisted of ART-experienced PLWH, 18 years of age or older, active in care (i.e., at least one clinic or telephone contact in the previous 12 months), and meeting either the HTE or non-HTE definitions on December 31, 2016, as defined below. The baseline regimen was defined as the regimen taken on December 31, 2016 and could have been initiated anytime on or before December 31, 2016. HTE and non-HTE PLWH were followed from initiation of their baseline regimen until the earliest of the following events: (a) loss to follow-up (i.e., 12 months after their last clinical contact), (b) death, or (c) study end (December 31, 2018). Additionally, for non-HTE PLWH who met the definition of HTE after December 31, 2016, follow-up was ended at the time the HTE criteria were first met.

### HTE definitions

Both HTE and non-HTE PLWH were identified based on their ART history and current ART regimen taken on December 31, 2016. The criteria for identifying HTE PLWH were based on preliminary work in OPERA, in which 2–11% of PLWH who were active in care were classified as HTE according to different definitions (Additional file 1: Fig. S1, Table S1) [9]. In the current study, HTE PLWH were defined as either (1) PLWH with exposure to at least three core agent classes prior to their baseline regimen, or (2) PLWH on a baseline regimen indicative of HTE. Core agent classes included non-nucleoside reverse transcriptase inhibitors (NNRTI), protease inhibitors (PI), integrase strand transfer inhibitors (INSTI), fusion inhibitors, and CCR5 antagonists. Regimens indicative of HTE contained either (a) dolutegravir with twice daily dosing; (b) darunavir with twice daily dosing; (c) etravirine; (d) both an INSTI and a PI; (e) maraviroc; or (f) enfuvirtide. Non-HTE PLWH were identified as PLWH who were ART-experienced, on a baseline regimen consisting of one core agent with two NRTIs, and who did not meet the definition of HTE described above; all other ART regimens were ineligible.

### Outcomes definitions

Achievement of virologic undetectability was defined as the first VL < 50 copies/mL during follow-up and assessed among viremic PLWH (i.e., baseline VL ≥ 50 copies/mL). Maintenance of virologic suppression was defined as maintaining a VL < 200 copies/mL throughout the remaining follow-up after achievement of virologic undetectability and was assessed among PLWH who were viremic at baseline. Virologic failure was defined as two consecutive VLs ≥ 200 copies/mL or one VL ≥ 200 copies/mL followed by core agent discontinuation and was assessed among PLWH who were virologically undetectable (i.e., baseline VL < 50 copies/mL). Immunologic preservation was defined as maintenance of a CD4 cell count ≥ 200 cells/µL throughout follow-up and was assessed among PLWH with a baseline CD4 cell count ≥ 200 cells/μL.

Regimen discontinuation was defined as either the switch, removal, or addition of a core agent, or over 45 days without an ART prescription. Incident morbidity was defined as either a new ADE or a new diagnosis of autoimmune disease, cardiovascular disease, invasive cancer, endocrine disorder, mental health disorder, liver disease, bone disorder, peripheral neuropathy, renal disease, or hypertension. For any given morbid condition, a prior diagnosis of the same morbid condition precluded its inclusion as an incident morbid condition, although a prior diagnosis of any other morbid condition did not.

### Statistical analysis

Baseline demographic and clinical characteristics were described among HTE and non-HTE PLWH using medians and interquartile ranges (IQR) for continuous variables and frequencies for categorical variables. Virologic outcomes were only ascertained among PLWH with at least one follow-up VL and immunologic outcomes were only ascertained among PLWH with at least one follow-up CD4 cell count. Regimen discontinuation, morbidity, and mortality were described among all PLWH in the study population. Time to each outcome was assessed using Kaplan–Meier methods, comparing HTE and non-HTE PLWH. Cumulative probabilities and 95% confidence intervals (CI) were estimated in each group at 12, 24 and 48 months after the baseline regimen initiation.

## Results

### Study population

The study population included 24,183 ART-experienced PLWH (Fig. 1). The criteria for HTE were met by 2277 PLWH (9%) who were followed for a median of 32 months (IQR 22, 50) from baseline regimen initiation until end of follow-up. Of those, 707 were identified as HTE by their treatment history (previously exposed to three or more core agent classes), 1497 were identified as HTE by currently being prescribed a regimen consistent with HTE, and 73 met both criteria for classification as HTE. The remaining 21,906 PLWH (91%) were considered non-HTE and were followed for a median of 28 months (IQR 20, 37) from baseline regimen initiation until end of follow-up.

At baseline, HTE PLWH tended to be older, had higher VLs, and lower CD4 cell counts compared to non-HTE PLWH. On average, HTE PLWH had been living with HIV longer, diagnosed a median of 15 years prior to December 31, 2016 (IQR 7, 22) compared with non-HTE PLWH who were diagnosed a median of 7 years prior (IQR 3, 15) (Table 1). HTE PLWH also experienced a higher burden of AIDS-defining conditions (54%), concomitant medications (65%), and morbid conditions (80%) compared to the non-HTE population (29%, 51%, and 69%, respectively) (Table 1, Additional file 1: Table S2).

**Table 1:** Baseline^a^ characteristics of heavily treatment-experienced (HTE) and non-heavily treatment-experienced (non-HTE) people living with HIV

	HTE population N = 2277	Non-HTE population N = 21,906
Demographic characteristics		
Age (yrs), median (IQR)	50 (42, 56)	44 (33, 52)
Sex, n (%)		
Female	431 (19)	3615 (17)
Male	1844 (81)	18,280 (83)
Unknown	2 (0)	11 (0)
Race, n (%)		
Black	906 (40)	8612 (39)
White	1239 (54)	11,693 (53)
Other	58 (3)	776 (4)
Unknown	74 (3)	825 (4)
Ethnicity, n (%)		
Hispanic	572 (25)	5626 (26)
Non-Hispanic	1644 (72)	15,813 (72)
Unknown	61 (3)	467 (2)
MSM, n (%)	1190 (52)	12,798 (58)
US geographic region, n (%)		
Northeast	109 (5)	2039 (9)
South	1189 (52)	11,267 (51)
Midwest	37 (2)	845 (4)
West	940 (41)	7755 (35)
US territories	≤ 5^b^	0 (0)
Clinical characteristics		
VL (copies/mL), n (%)		
Median (IQR)	82 (19, 15,650)	19 (19, 100)
< 50	900 (40)	14,645 (67)
50 to < 200	209 (9)	1729 (8)
≥ 200 to < 10,000	331 (15)	1856 (9)
≥ 10,000 to < 100,000	345 (15)	1957 (9)
≥ 100,000	202 (9)	818 (4)
Missing	290 (13)	901 (4)
CD4 cell count (cells/µL), n (%)		
Median (IQR)	412 (209, 636)	587 (396, 801)
> 500	761 (33)	13,007 (59)
> 350 to ≤ 500	402 (18)	3833 (18)
> 200 to ≤ 350	345 (15)	2505 (11)
> 50 to ≤ 200	337 (15)	1299 (6)
≤ 50	146 (6)	355 (2)
Missing	286 (13)	907 (4)
Years since HIV diagnosis, median (IQR)	15 (7, 22)	7 (3, 15)
Year of ART initiation, median (IQR)	2012 (2005, 2015)	2013 (2011, 2015)
History of ADE^c^, n (%)	1221 (54)	6294 (29)
Morbid conditions^d^	1823 (80)	15,132 (69)
VACS mortality risk^e^, median (IQR)	24 (13, 41)	12 (6, 23)
Concomitant medications^f^	1477 (65)	11,071 (51)

*ADE* AIDS defining event; *ART* antiretroviral therapy; *HTE* heavily treatment-experienced; *IQR* interquartile range; *mL* milliliter; *MSM* men who have sex with men; *n* number; *N/A* not available; *µL* microliter; *US* United States; *VACS* Veterans Aging Cohort Score; *VL* viral load; *yrs* years

^a^Baseline defined as the start of the regimen taken on December 31, 2016

^b^HIPAA requires the masking of cells with 1 to 5 individuals

^c^History of ADE refers to the time period at or prior to baseline

^d^Diagnosis of autoimmune disease, cardiovascular disease, invasive cancer, endocrine disorder, mental health disorder, liver disease, bone disorder, peripheral neuropathy, renal disease, or hypertension

^e^VACS Mortality Index: Scored by summing pre-assigned points for age, CD4 count, HIV-1 RNA, hemoglobin, platelets, aspartate and alanine transaminase, creatinine, and viral hepatitis C infection. A higher score is associated with a higher risk of 5-year all-cause mortality [17]

^f^Direct acting antivirals, antidepressants, non-steroidal anti-inflammatory agents, immune modulators, antibiotics, anxiolytics/hypnotics/sedatives, lipid lowering agents, anti-diabetics

### Virologic outcomes

**Fig. 2:**
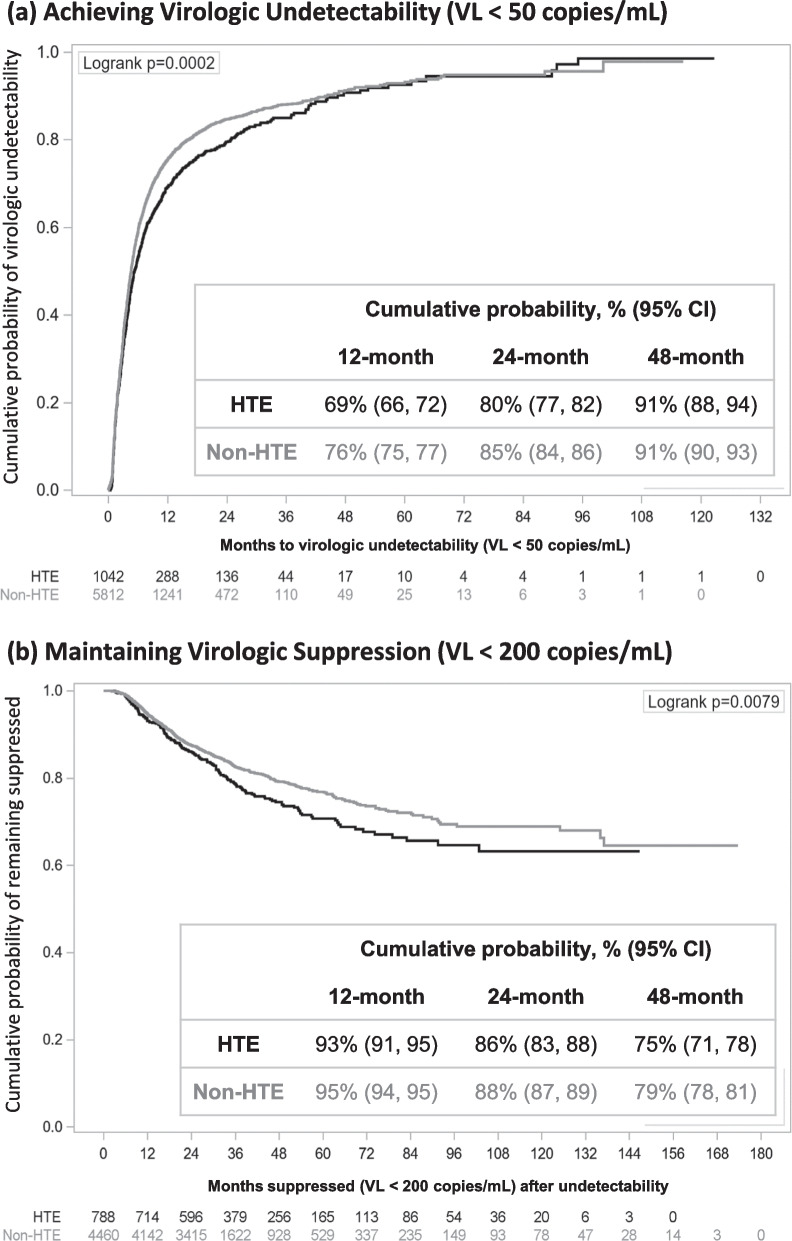
Cumulative probability of (**a**) achieving virologic undetectability to viral load (VL) < 50 copies/mL among viremic people living with HIV (PLWH) (VL ≥ 50 copies/mL) at baseline and (**b**) maintaining virologic suppression to VL < 200 copies/mL among viremic PLWH at baseline who achieved virologic undetectability (VL < 50 copies/mL) over follow-up

Virologic outcomes were assessed in the subset of the study population with at least one VL measured during follow-up. Among 1042 HTE and 5812 non-HTE PLWH who were viremic (VL ≥ 50 copies/mL) at baseline and had follow-up VLs, HTE PLWH had lower cumulative probabilities of achieving undetectability (VL < 50 copies/mL) at 12 and 24 months than non-HTE PLWH, although no difference between the two groups was observed at 48 months (Fig. 2a). At 24 months, the cumulative probability of virologic undetectability was estimated at 80% (95% CI 77, 82) among HTE PLWH and 85% (95% CI 84, 86) among non-HTE PLWH.

Among the 788 HTE and 4460 non-HTE PLWH who achieved undetectability (VL < 50 copies/mL) during follow-up and had additional follow-up VLs, there was no difference in cumulative probabilities of maintaining suppression (VL < 200 copies/mL) at 12, 24, and 48 months; all confidence intervals overlapped. However, despite overlapping confidence intervals, the numeric probability of maintaining suppression appeared to drop more rapidly among HTE than non-HTE PLWH (Fig. 2b).

**Fig. 3:**
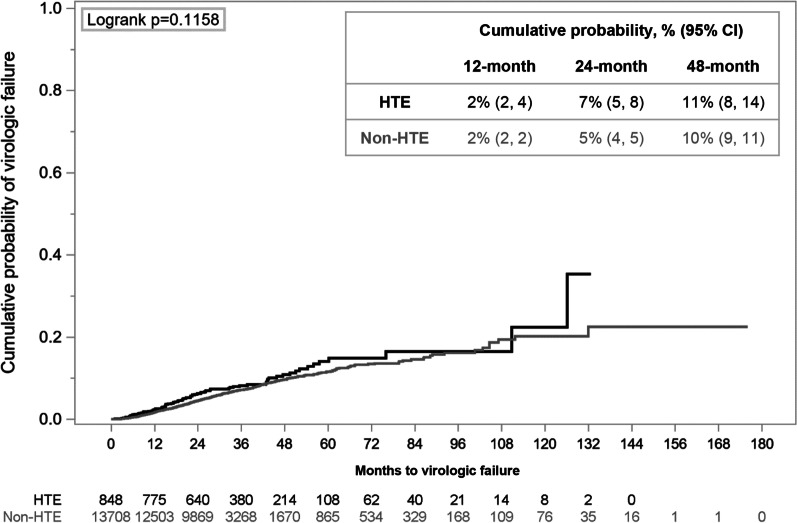
Cumulative probability of virologic failure (two consecutive viral loads ≥ 200 copies/mL or discontinuation following a viral load ≥ 200 copies/mL) among people living with HIV with a viral load < 50 copies/mL at baseline

Among 848 HTE and 13,708 non-HTE virologically undetectable PLWH (VL < 50 copies/mL) at baseline with follow-up VLs, no difference in the cumulative probability of virologic failure was observed between groups, which remained low throughout follow-up (24 months: 7% HTE, 5% non-HTE) (Fig. 3).

### Immunologic preservation

**Fig. 4:**
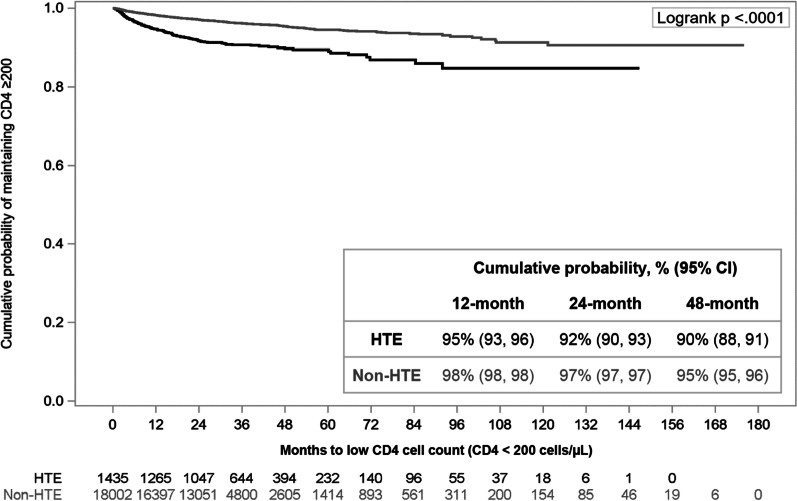
Cumulative probability of maintaining CD4 cell count ≥ 200 cells/µL among people living with HIV with CD4 cell count ≥ 200 cells/µL at baseline

Most PLWH initiated their baseline regimen with a CD4 cell count ≥ 200 cells/µL (HTE: 66%; non-HTE: 88%); 1435 HTE and 18,002 non-HTE PLWH had follow-up CD4 cell counts. The majority maintained CD4 cell counts ≥ 200 cells/µL throughout follow-up among both the HTE (n = 1299, 91%) and the non-HTE PLWH (n = 17,360, 96%). Nevertheless, the cumulative probability of immunologic preservation was lower among HTE compared to non-HTE PLWH throughout follow-up, with 24-month cumulative probabilities of 92% (95% CI 90, 93) for HTE and 97% (95% CI 97, 97) for non-HTE PLWH (Fig. 4).

### Regimen discontinuation

Discontinuations (i.e., switch, removal or addition of a core agent) of HTE baseline regimens were frequent (45%) and occurred a median of 27 months (IQR 17, 46) after initiation. In comparison, discontinuations of non-HTE baseline regimens occurred slightly less frequently (41%) but sooner (median 23 months, IQR 14, 34). HTE regimen discontinuations were more likely to include a switch of one or more core agents than the non-HTE regimens. The non-HTE regimens were more likely to include a break from all antiretrovirals before restarting therapy (Table 2).

**Table 2:** Regimen discontinuations in heavily treatment-experienced (HTE) and non-heavily treatment-experienced (non-HTE) people living with HIV

	HTE population N = 2277	Non-HTE population N = 21,906
Discontinuations, n (%)	1032 (45)	9049 (41)
Months to discontinuation, median (IQR)	27 (17, 46)	23 (14, 34)
Type of discontinuation, n (%)		
Core agent class switch (single baseline class)	81 (8)	3,241 (36)
Same core agent class (single baseline class)	50 (5)	2055 (23)
Multiple core agent classes switched	603 (58)	0 (0)
Treatment gap > 45 days	214 (21)	3023 (33)
No following regimen	84 (8)	730 (8)

*HTE* heavily treatment-experienced; *IQR* interquartile range; n, number

### Incident morbidities and mortality

Over follow-up, incident ADEs were uncommon, but more frequent among HTE PLWH (n = 108, 5%) than non-HTE PLWH (n = 506, 2%). However, median time to diagnosis of incident ADEs did not differ between the HTE (13 months, IQR 4, 29) and non-HTE groups (12 months, IQR 5, 23). Incident non-ADE morbidities were common in both populations, though more likely and occurring sooner among HTE PLWH (n = 1026, 45%; median 10 months, IQR 3, 22) than non-HTE PLWH (n = 7608, 35%; median 11 months, IQR 5, 22). Deaths were uncommon among HTE (n = 36, 2%) and non-HTE (n = 163, 1%) PLWH. The median time to death was longer in the HTE group (30 months, IQR 19, 36) than in the non-HTE group (21 months, IQR 15, 33).

## Discussion

In the OPERA® cohort, the 2277 PLWH identified as HTE on December 31, 2016 tended to be older, had higher VLs, lower CD4 cell counts as well as a higher burden of ADEs, concomitant medications and non-ADE morbid conditions than the 21,906 non-HTE PLWH. HTE PLWH experienced more unfavorable treatment outcomes than non-HTE PLWH in some regards. They were numerically less likely to remain virologically suppressed (VL < 200 copies/mL) and maintain their CD4 cell counts above 200 cells/µL over follow-up. They were also more likely to develop new morbidities than non-HTE PLWH. However, at 24 months after regimen start, HTE PLWH still had an 80% cumulative probability of achieving undetectability (VL < 50 copies/mL) and a 92% cumulative probability of maintaining CD4 cell counts ≥ 200 cells/µL. In addition, most HTE PLWH were still on their baseline regimen at study end, and fewer than 2% died over the course of follow-up.

As shown in other observational studies [10, 13, 15, 18, 19], HTE PLWH in OPERA® experienced less favorable clinical outcomes than non-HTE PLWH. In terms of virologic response, viremic HTE PLWH had lower cumulative probabilities of achieving undetectability (VL < 50 copies/mL), although once achieved, no difference was detected in the likelihood of maintaining suppression (VL < 200 copies/mL) or of virologic failure between HTE and non-HTE PLWH. Among 10,237 virologically suppressed PLWH in the UK CHIC Study, the risk of viral rebound (i.e., two VLs > 400 copies/mL) increased with each previously failed ART regimen. Within the 1st year after viral suppression, the rate of viral rebound was 33 per 100 person-years (95% CI 28, 38) among PLWH who had failed at least four regimens; these HTE PLWH were more than four times more likely to experience viral rebound than non-HTE who had not previously failed an ART regimen (adjusted incidence rate ratio [aIRR] 4.69, 95% CI 3.61, 4.73) [18]. In a study of 247 PLWH who had failed at least three ART regimens including ARVs from three classes, the risk of viral rebound decreased with each additional ARV in the current regimen which had not been previously failed (risk ratio [RR]: 0.78 per additional ARV; 95% CI 0.65, 0.95) [13].

While the cumulative probability of immunologic preservation in OPERA® was lower among HTE than non-HTE PLWH, it is noteworthy that over 90% maintained CD4 cell counts ≥ 200 cells/µL throughout follow-up. Moreover, the discrepancies between the virologic and immunologic findings in this study are of interest. Once virologic undetectability was achieved, maintenance of suppression was similar between the HTE and non-HTE groups, yet HTE PLWH were less likely to maintain CD4 cell counts ≥ 200 cells/µL than non-HTE PLWH. In contrast, over 6 months of follow-up in the Swiss HIV Cohort Study, CD4 cell counts tended to stabilize or increase despite VLs remaining ≥ 500 copies/mL among most of 23 heavily pre-treated PLWH, although no comparisons to non-HTE PLWH were made in this very small study [15]. The discordance between virologic and immunologic outcomes observed in OPERA® may be explained in part by the fact that HTE PLWH had been living with HIV for longer at the time of the study, a median of 15 years for HTE and 7 years for non-HTE PLWH. Potent ART regimens were able to reduce VLs but may have been insufficient for immunologic preservation in the HTE context. Newer and future agents demonstrating CD4 improvements such as ibalizumab, fostemsavir, and lenacapavir may therefore have an important impact in the HTE regimen landscape [20–22].

In OPERA®, incident ADEs and death were more common among HTE than non-HTE PLWH, although both occurred infrequently. Similarly, in a 2004 EuroSIDA cohort analysis of 3496 PLWH, the incidence of new cases of AIDS or death among PLWH with triple drug-class failure (5 per 100 person-years) was nearly double that of PLWH without triple drug-class failure (2.7 per 100 person-years) [19]. In addition, among 15,570 PLWH in a 2021 analysis of the EuroSIDA cohort, HTE PLWH experienced higher incidence rates of new ADEs and non-ADE morbidities than non-HTE PLWH. However, after adjusting for age, CD4 count, and prior ADEs, HTE status was no longer associated with the development of a new ADE (aIRR 1.44, 95% CI 0.86, 2.40) or new non-ADE morbidity (aIRR 0.96, 95% CI 0.74, 1.25) [10].

This study is not without limitations. In the absence of resistance data, there is no standard definition for classifying PLWH as HTE and each definition of HTE has the potential to misclassify treatment experience. In observational studies where resistance data are unavailable, such as this OPERA® study, an approach based on prior ARV exposure or virologic failure has generally been favored [9, 13, 23, 24]. In the OPERA® setting, a two-component definition of HTE was deemed preferable in preliminary work, balancing limitations of each definition (i.e., PLWH with exposure to at least three core agent classes prior to their baseline regimen, or PLWH on a baseline regimen indicative of HTE; Additional file 1: Fig. S1, Table S1) [9]. Identifying HTE PLWH based on prior ARV exposure to three or more core agent classes rather than based on history of treatment failures may have overestimated the number of HTE PLWH in this study. In contrast, looking only at prior virologic failures misses individuals whose treatment options may be limited, in part, due to potential drug–drug interactions, or toxicities with certain regimens. An HTE definition based only on previous ART experience requires access to full, detailed history of specific ART regimens used, which may not be available for patients who transferred to an OPERA® clinic after ART initiation, potentially underestimating the number of HTE PLWH in this study. However, identifying PLWH on a regimen indicative of HTE did not rely on historical data as it was based on the current regimen. With the majority of HTE PLWH in this study identified because their current regimen was indicative of HTE (n = 1570, 70%), misclassification is likely limited to a minority of PLWH. As many as a quarter of PLWH with exposure to three or more core agent classes were on their 10th or later line of ART (24%). While 60% of those on a regimen indicative of HTE had experienced only two classes of ART core agents, including their current regimen, the regimens selected are not recommended as first line therapy and are therefore likely identifying PLWH with incomplete medication histories recorded in their EHR. With potential misclassification of PLWH as HTE both over- and under-estimating the prevalence of HTE, the direction of potential bias in the estimates of the effect of HTE on clinical outcomes is difficult to predict.

Another limitation of this study stems from the fact that reasons for treatment discontinuation are not well-captured in most EHR data sources, including the OPERA® cohort. Therefore, in most cases, it is impossible to determine whether the baseline regimen or any prior regimen was discontinued due to failure, poor tolerability, safety issues, or other reasons such as transitioning to newer classes, formulary changes, or simplification (e.g., switching to single tablet regimens). Discontinuations driven by factors other than treatment effectiveness may thus have contributed to an overestimation of HTE PLWH in our study population. Finally, this study was descriptive in nature, and therefore, no statistical adjustments were made to control for confounding. However, the study was able to provide a thorough description of HTE PLWH and their treatment outcomes.

Strengths of this study include the use of the OPERA® cohort, a large database of the EHR data for 94,852 PLWH from 79 locations across 15 states in the US and Puerto Rico at the time of this study. The OPERA® cohort included approximately 8% of all PLWH in care in the US at the time of this study [25]. In addition, participating clinics vary in size, setting (rural, urban), and specialization (general care, infectious disease specialty). The results of this study are thus a good representation of HIV care in the US. The use of EHR data also allowed for a real-world assessment of treatment experience and long-term outcomes in the US, with an overall median follow-up of 28 months (IQR 21, 38). Finally, with follow-up through 2018, this study is the most recent of only a few observational studies comparing clinical outcomes between HTE and non-HTE PLWH in the absence of resistance data; one study included data up to 2003 only [19] and another included data from 2010 to 2016 [10].

## Conclusion

In this large and representative US-based cohort, HTE PLWH were characterized by a complex clinical presentation, representing an older population with a greater morbidity burden than non-HTE PLWH. Furthermore, compared to non-HTE PLWH, HTE PLWH were at greater risk of unfavorable virologic and immunologic outcomes such as a lower likelihood of viral undetectability or immunologic preservation. Given the less favorable treatment outcomes among HTE PLWH, additional therapeutic options are needed for this vulnerable population.

## Supplementary Information

**Additional file 1: Figure S1.** Counts of people living with HIV (PLWH) by various heavily treatment-experienced (HTE) definitions and their overlap, out of the total number of PLWH in care on December 31, 2016 (n = 41,939). **Table S1.** ART experience among people living with HIV by various heavily treatment-experienced definitions^a^. **Table S2.** Comorbid conditions and concomitant medications at baseline among heavily treatment-experienced (HTE) and non-heavily treatment-experienced (non-HTE) people living with HIV

## Data Availability

The datasets used in this study are not publicly available due to privacy concerns and the proprietary nature of the database but can be accessed upon reasonable request through the corresponding author to the OPERA® Epidemiological and Clinical Advisory Board.

## Abbreviations

ADE: AIDS defining event
aIRR: Adjusted incidence rate ratio
ART: Antiretroviral therapy
ARV: Antiretrovirals
CI: Confidence interval
EHR: Electronic health records
HIPAA: Health Insurance Portability and Accounting Act
HITECH: Health Information Technology for Economic and Clinical Health Act
HTE: Heavily treatment-experienced
INSTI: Integrase strand transfer inhibitor
IQR: Interquartile range
IRB: Institutional review board
NNRTI: Non-nucleoside reverse transcriptase inhibitor
NRTI: Nucleoside reverse transcriptase inhibitors
OPERA®: Observational Pharmacoepidemiology Research and Analysis
PI: Protease inhibitor
PLWH: People living with HIV
RR: Risk ratio
UK: United Kingdom
US: United States
VL: Viral load
