# Supplementary material for: Heavily treatment-experienced people living with HIV in the OPERA® cohort: population characteristics and clinical outcomes
Source: BMC Infect Dis. 2023 Feb 13;23:91. doi: 10.1186/s12879-023-08038-w (PMC9926692; doi:10.1186/s12879-023-08038-w)
Supplement: Supplementary file 1 — Additional file 1: Figure S1. Counts of people living with HIV (PLWH) by various heavily treatment-experienced (HTE) definitions and their overlap, out of the total number of PLWH in care on December 31, 2016 (n = 41,939). Table S1. ART experience among people living with HIV by various heavily treatment-experienced definitionsa. Table S2. Comorbid conditions and concomitant medications at baseline among heavily treatment-experienced (HTE) and non-heavily treatment-experienced (non-HTE) people living with HIV [file 12879_2023_8038_MOESM1_ESM.docx]

**ADDITIONAL TABLES & FIGURES**

**Heavily Treatment-Experienced People Living with HIV in the OPERA® Cohort: Population Characteristics and Treatment Outcomes**

Ricky K. Hsu^1,2^, Jennifer S. Fusco^3^, Cassidy E. Henegar^4^, Vani Vannappagari^4^, Andrew Clark^5^, Laurence Brunet^3^, Philip C. Lackey^6^, Gerald Pierone Jr.^7^, Gregory P. Fusco^3^

^1^ NYU Langone Health Center, New York, NY, USA

^2^ AIDS Healthcare Foundation, New York, NY, USA

^3^ Epividian, Inc., Durham, NC, USA

^4^ ViiV Healthcare, Research Triangle Park, NC, USA

^5^ ViiV Healthcare, Brentford, Middlesex, UK

^6^ Signature Healthcare, Charlotte, NC, USA

^7^ Whole Family Health Center, Vero Beach, FL, USA

**
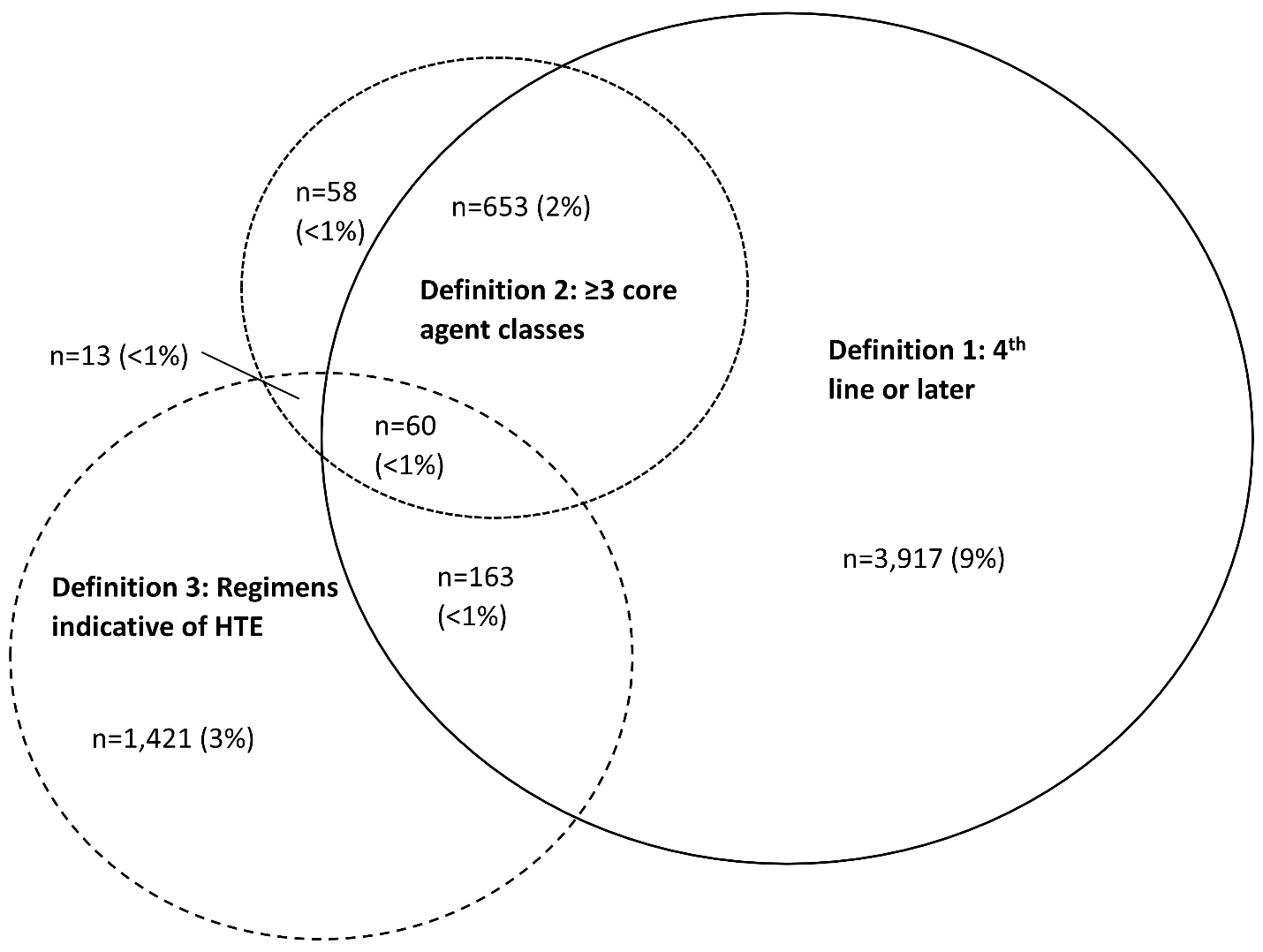
**

**Additional Figure S1. Counts of people living with HIV (PLWH) by various heavily treatment-experienced (HTE) definitions and their overlap, out of the total number of PLWH in care on December 31, 2016 (n=41,939).** This figure represents preliminary work that informed the eventual definitions of HTE and non-HTE populations in the main analyses of this manuscript. Definition 1 included PLWH on their fourth line or later of antiretroviral therapy (ART) at their baseline regimen, where a new line of ART was determined as any switch in core agent (i.e., any non-nucleoside reverse transcriptase inhibitor). Definition 2 included PLWH with exposure to at least three core agent classes prior to their baseline regimen. Definition 3 included PLWH on a baseline regimen indicative of HTE; regimens included either (a) dolutegravir twice daily; (b) darunavir twice daily; (c) etravirine; (d) an integrase strand transfer inhibitor and a protease inhibitor; (e) maraviroc; or (f) enfuvirtide. The estimated proportion of PLWH referred to as HTE by Definition 1 was 11.4% (n=4,793 [95% CI: 11.1%, 11.7%]); by Definition 2 was 1.9% (n=784 [95% CI: 1.7%, 2.0%]); and by Definition 3 was 4.0% (n=1,657 [95% CI: 3.8%, 4.1%]).

**Additional Table S1. ART experience among people living with HIV by various heavily treatment-experienced definitions^a^**

|  | **Definition 1: Currently on ≥4^th^ of ART^b^**  **n=4,793** | **Definition 2: Exposure to ≥3 core agent classes^c^**  **n=784** | **Definition 3: Specific ART regiments indicative of HTE^d^**  **n=1,657** |
| --- | --- | --- | --- |
| Current line of ART, n (%) |  |  |  |
| 4^th^ or lower line | 1972 (41) | 166 (21) | 1,485 (90) |
| 5^th^ line | 1065 (22) | 100 (13) | 50 (3) |
| 6^th^ line | 620 (13) | 118 (15) | 30 (2) |
| 7^th^ line | 402 (8) | 71 (9) | 23 (1) |
| 8^th^ line | 250 (5) | 80 (10) | 20 (1) |
| 9^th^ line | 162 (3) | 62 (8) | 16 (1) |
| 10^th^ or higher line | 322 (7) | 187 (24) | 33 (2) |
|  |  |  |  |
| Months since ART initiation, median (IQR) | 111 (69, 191) | 142 (90, 206) | 33 (11, 74) |
|  |  |  |  |
| Core agent classes ever, n (%) |  |  |  |
| 1 | 501 (11) | 0 (0) | 159 (10) |
| 2 | 2036 (43) | 0 (0) | 825 (50) |
| 3 | 2044 (43) | 641 (82) | 604 (37) |
| 4 | 197 (4) | 130 (17) | 64 (4) |
| 5 or more | 15 (< 1) | 13 (2) | 5 (< 1) |
|  |  |  |  |
| 2 or more core agents in current regimen, n (%) | 1,562 (33) | 379 (51) | 1,464 (88) |

ART, antiretroviral therapy; HTE, heavily treatment-experienced; IQR, interquartile range; n, number.

^a^ This table represents preliminary analyses among 41,939 adults living with HIV who were active in care on 31DEC2016.

^b^ Change in line of ART is denoted by a change in core agent.

^c^ Initiated and discontinued core agents from at least three separate classes.

^d^ PLWH taking a regimen consisting of either: dolutegravir, twice daily; darunavir, twice daily; etravirine; an integrase strand transfer inhibitor and protease inhibitor; maraviroc; or enfuvirtide.

**Additional Table S2. Comorbid conditions and concomitant medications at baseline among heavily treatment-experienced (HTE) and non-heavily treatment-experienced (non-HTE) people living with HIV**

|  | **HTE Population**  **N=2,277** | **Non-HTE Population**  **N=21,906** |
| --- | --- | --- |
| **Comorbid conditions, n (%)** | 1823 (80) | 15132 (69) |
| Autoimmune Disease | 77 (3) | 543 (3) |
| Cardiovascular Disease | 374 (16) | 1,909 (9) |
| Invasive Cancers | 253 (11) | 1,281 (6) |
| Endocrine Disorders | 1,047 (46) | 7,604 (35) |
| Mental Health Disorders | 833 (37) | 6,707 (31) |
| Liver Disease | 541 (24) | 3,721 (17) |
| Bone Disorders | 94 (4) | 470 (2) |
| Peripheral Neuropathy | 426 (19) | 1,836 (8) |
| Renal Disease | 392 (17) | 1,832 (8) |
| Hypertension | 827 (36) | 6,199 (28) |
| **Concomitant medications, n (%)** | 1477 (65) | 11071 (51) |
| Direct Acting Antivirals | 10 (<1) | 156 (1) |
| Antidepressants | 571 (25) | 3,967 (18) |
| Non-steroidal Anti-inflammatory Agents | 264 (12) | 1,845 (8) |
| Immune Modulators | 259 (11) | 1,919 (9) |
| Antibiotics | 366 (16) | 2,365 (11) |
| Anxiolytics/Hypnotics/Sedatives | 392 (17) | 2,565 (12) |
| Lipid lowering agents | 595 (26) | 3,641 (17) |
| Anti-diabetics | 203 (9) | 1,130 (5) |

ART, antiretroviral therapy; HTE, heavily treatment-experienced; n, number.
